# Mechanism simulation of polar and nonpolar organic solvent vapor adsorption on a multiwall carbon nanotubes paper gas sensor†

**DOI:** 10.1039/d4ra04474f

**Published:** 2024-08-09

**Authors:** Mengli Zhang, Shuhei Inoue, Yukihiko Matsumura

**Affiliations:** a Graduate School of Advanced Science and Engineering, Hiroshima University Higashi-Hiroshima 739-8527 Japan zml@hiroshima-u.ac.jp; b Department of Mechanical Engineering, Kindai University Higashi-Hiroshima 739-2116 Japan inoue@hiro.kindai.ac.jp; c Graduate School of Advanced Science and Engineering, Hiroshima University Higashi-Hiroshima 739-8527 Japan mat@hiroshima-u.ac.jp

## Abstract

We investigate the adsorption behavior of polar and nonpolar molecules on carbon nanotube interfaces through computational simulations. Gaussian 16 was utilized to calculate the total energy of each possible molecular structure and analyze the adsorption mechanisms in stacked and inline configurations. The study reveals that nonpolar molecules favor stacked adsorption on two graphene interfaces, while polar molecules prefer inline adsorption. The findings suggest that inline adsorption of polar molecules results in minimal changes to the local dielectric constant, which may explain the absence of multi-step adsorption isotherms. The research examines the stability and energetics of molecular adsorption on graphene layers simulating CNT interfaces. Different types of molecules (polar and nonpolar) exhibit distinct adsorption behaviors, with nonpolar molecules aligning with the IUPAC type VI isotherm model and polar molecules following the Langmuir isotherm model (IUPAC type I). This study provides insight into how molecules are likely to adsorb on CNT surfaces and the impact on the local dielectric constant. This understanding has implications for the design and optimization of CNT-based sensors, particularly in detecting organic solvents and gases in various environments.

## Abstract 2

For the purpose of development of CNT based gas sensor, we clarified the different behaviour depending on polarity shown in adsorption isotherm.

## Graphical abstract

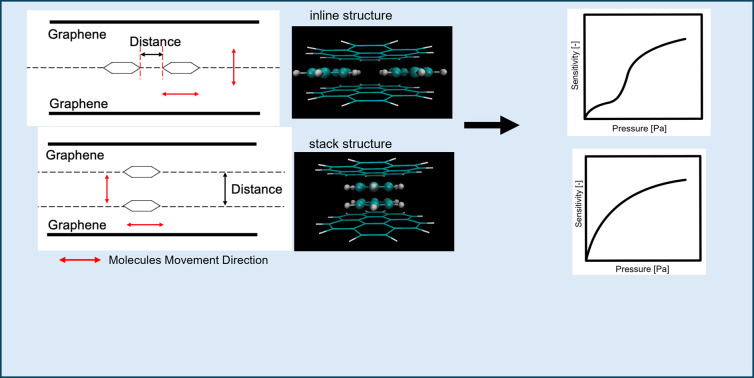

## Introduction

Gas sensors are essential for everyday life and have been applied in various fields such as the chemical industry,^1,2^ power plants, households, environmental monitoring,^3–5^ and more due to their ability to detect the type and concentration of toxic or multiple gases in the environment.^6,7^

Every year, several incidents involving the leakage of poisonous organic solvents occur; therefore, producing sensors that can rapidly identify organic solvents is crucial. Respirators with filters that are replaced at the end of their lifespan are commonly used when working with organic solvents for safety reasons. However, the filter's lifespan is short and is not specified for each type of gas. To ensure worker safety, filters are often replaced earlier than required, which increases costs.^8–10^

Real-time measurement of organic solvents is necessary because the volume of gas inhaled varies from person to person, and the concentration of organic solvents depends on the environment. Moreover, some gases can leak without smell or colour, creating a dangerous situation for the worker.^11–13^

Carbon nanotubes (CNTs) are well-suited for creating gas sensors with structures and components that exhibit novel and significantly enhanced physical and chemical properties due to their nanoscale size. CNTs, which have wall structures made from a single graphite sheet rolled into a tubular shape, are known as single-walled carbon nanotubes (SWCNTs). In contrast, those consisting of multiple graphite sheets, each rolled into a tubular shape and layered one within the other, are known as multi-walled carbon nanotubes (MWCNTs). Due to its tubular shape, a carbon nanotube has a length that can exceed one hundred to several thousand times its diameter.^14^

Many theoretical and simulation studies have been conducted to understand carbon nanotubes and related phenomena.^15–17^ Experimental studies have revealed carbon nanotubes' unique properties in electrical, physical, chemical, and mechanical aspects.^18–25^ They hold significant potential for generating various valuable equipment and applications such as sensors, electronic devices, batteries, field emitters, and hydrogen storage.^26–32^ Despite extensive research on CNT-based gas detectors, the sensor's ability to detect organic solvents remains inadequate. Gas molecules are primarily divided into two main categories: polar and nonpolar molecules. Researchers typically use Lewis structures to determine whether a molecule is polar or nonpolar. Nonpolar compounds tend to be symmetric, with all sides around the central atom identically bonded to the same element and no unshared pairs of electrons. Polar molecules, on the other hand, are asymmetric, containing lone pairs of electrons on a central atom or having atoms with different electronegativities bonded together. Each gas behaves differently when adsorbing onto the gas sensor, primarily depending on its polar and nonpolar properties, such as NH_3_, H_2_, NO_2_, CO, and CO_2_.^33,34^

Polar and nonpolar organic molecules exhibit different behaviours when graphene adsorbs them. For polar molecules, they exhibit one-layer adsorption, which aligns with the Langmuir adsorption isotherm model. In contrast, nonpolar molecules exhibit step-like behaviour and align well with IUPAC type VI isotherm model. However, the underlying adsorption mechanism for polar and nonpolar molecules remains unclear.^35^ Similar behaviour was observed in the adsorption onto CNT film.^36^ This reported response mechanism of the CNT film^37^ suggests that the response stems from changes in electric resistance, which should exhibit a Langmuir-like isothermal curve. Given that bimolecular adsorption takes place in the high concentration range, the structure between adsorbed molecules is anticipated to influence the alteration in electrical resistance of the CNT thin film. Investigating this mechanism is essential for further developing gas sensors. There is minimal research on this point; thus, the novelty of this research lies in investigating how a second layer of molecules adsorbs onto a graphene surface after the first layer is already adsorbed. The study explores whether two adsorbate molecules form a linear connection between graphene sheets or stack on top of each other, focusing on which configuration is more energetically stable in carbon nanotubes. This work uses Gaussian 16 to calculate the total energy of each possible structure. The structure with the lowest adsorption energy consumption is the one most likely to be adopted by the molecules due to its chemical stability.

**Fig. 1:**
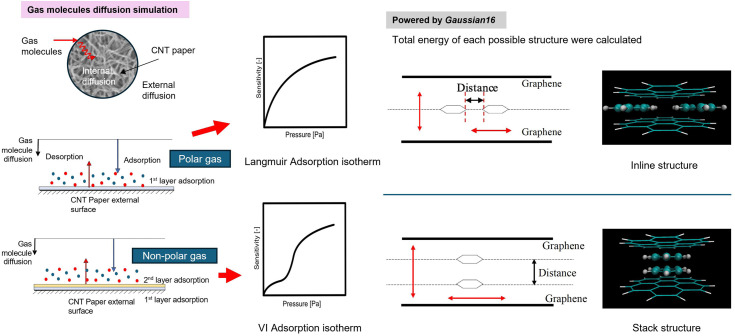
Schematics of polar and nonpolar molecules adsorption behaviour. In fact, molecules adsorbed onto the intersecting carbon nanotube interfaces are considered to adsorb at adsorption sites, so the adsorption layer does not become two layers as shown in the figure. However, in the adsorption of polar molecules, behaviour such as monolayer adsorption is observed, while in the adsorption of non-polar molecules, behaviour resembling bilayer adsorption appears on the adsorption isotherm. Gaussian 16 was used to calculate the total energy of each structure to determine if two adsorbate molecules form a linear connection between two graphene sheets or stack on top of each other.

The hypothesis of molecule adsorption behaviour according to our experimental and model fitting is shown in Fig. 1. For polar molecules, the results satisfy the Langmuir adsorption isotherm model (IUPAC I), which entails one-layer adsorption. Meanwhile, for nonpolar molecules, they exhibit step-like behaviour and align well with the IUPAC VI isotherm model, suggesting two-layer adsorption. This work proposes an inline structure and a stacked structure (shown in Fig. S1 and S2 of ESI,† respectively) as the basis for the calculation model. Carbon nanotube film is not flat, as depicted in the model in Fig. 1; instead, it is entangled in a spaghetti-like manner. Therefore, it's essential to focus on the adsorption occurring at the interface of two carbon nanotubes in real-world scenarios.

## Methods

Gaussian 16 (using the HF method with the 6-31G basis set) was utilized to calculate the total energy of each possible structure. In the actual adsorption model, the graphene surface is effectively infinite compared to the size of the adsorbing molecules. Considering an endless graphene model in our calculations would be impractical due to the vast number of required computations. Thus, to model the arrangement of the second layer of molecules on the graphene surface after the first layer has been adsorbed, we employed a finite model of graphene comprising several separate carbon rings. Given the size of the adsorbed molecules and the scope of our calculations, we used seven carbon rings in our simulation as a representative model of graphene. The total energy of two layers of graphene, as calculated by Gaussian 16, is 7.982 fJ.

Regarding the adsorption phases at the interface of carbon nanotubes, we examined two simple models. As mentioned earlier, for simplicity, carbon nanotubes are represented by graphene sheets. The discussion centres around whether two adsorbate molecules form a linear connection between two graphene sheets or whether the adsorbate molecules stack on top of each other. The focus is on determining which configuration is energetically more stable.

The carbon nanotubes used in the experiment are multi-walled carbon nanotubes with a specified diameter ranging from 20 to 40 nm. From the perspective of the adsorbate molecules, the curvature is not perceived due to this diameter range, making it appropriate to substitute them with a planar structure. In actual adsorption, it is unclear whether the adsorbate molecules attach along a line connecting the centres of the cross-sections of the two carbon nanotubes or if they attach slightly away from that line.

This study aims to determine the most stable adsorption position by optimizing the distance between the two graphene layers when the adsorbate molecules are present. We are not exploring rotated configurations for non-planar structures such as methanol and are only considering distances from one direction.

## Results and discussion

### Total energy of one nonpolar molecule

**Fig. 2:**
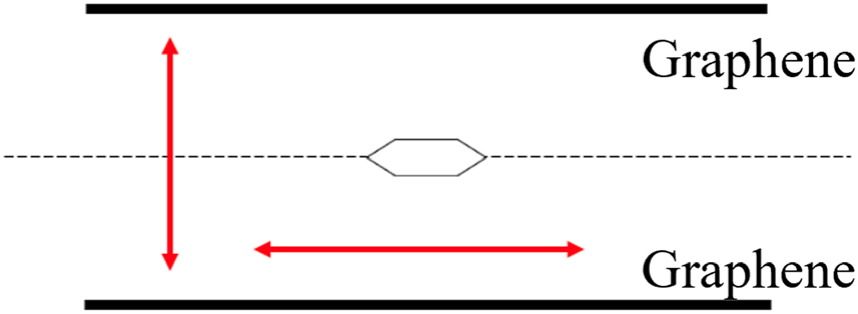
The possible direction of the benzene molecule and graphene and the red arrow represents the possible moving direction of molecules and graphene.

**Fig. 3:**
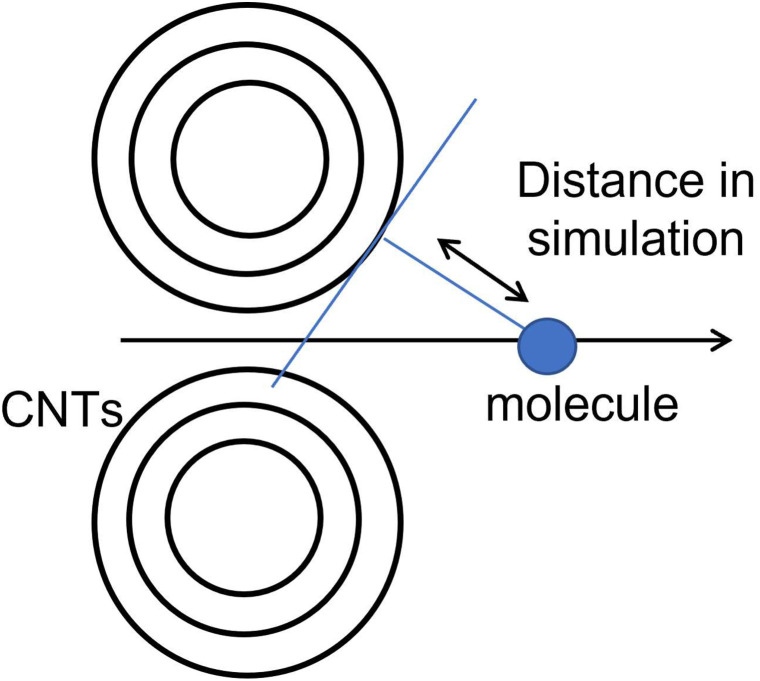
The relationship between graphene spacing and the distance of adsorption to CNTs.

We chose benzene as our sample of nonpolar molecules. This choice is due not only to its simple structure but also because it is one of the most commonly used organic solvents. The total energy of one benzene molecule, as calculated by Gaussian 16, was 1.005 fJ. This study also examined the effect of the molecule's position and the distance between two graphene layers on the system's energy. To investigate the effect of the molecule's position, we used the symmetry *x*-axis as a reference and altered the molecule's position from the centre to the edge of the graphene; the values of the symmetry *x*-axis used were 0, 54.45, 108.9, 399.3, 544.5, and 834.9 pm. The results are listed in Table 1. The distance between the two graphene layers varied by decreasing or increasing the distance, as shown in Fig. 2. The minimum energy in this structure occurred at the third location with the symmetry *x*-axis at 108.9 pm (ESI, Fig. S3†). The distance between the graphene layers was varied to 290.40, 290.00, 289.60, and 284.60 pm. The effect of the distance change in graphene is overall less significant than the change in location, resulting in nearly no energy change (shown in Fig. S4 in the ESI Section†). In actual adsorption phenomena, as mentioned earlier, the structure involved is not graphene but CNTs. The difference in energy due to the distance between graphene layers in simulations, as shown in Fig. 3, actually indicates how molecules adsorb at positions away from CNTs to achieve stabilization.

**Table 1:** Energy changes with the location of one benzene molecule model

Symmestry axis *x* [pm]	Total energy [fJ]
0	8.976
54.45	8.975
108.9	8.972
399.3	8.979
544.5	8.983
834.9	8.988

### Total energy of two nonpolar molecules

**Fig. 4:**
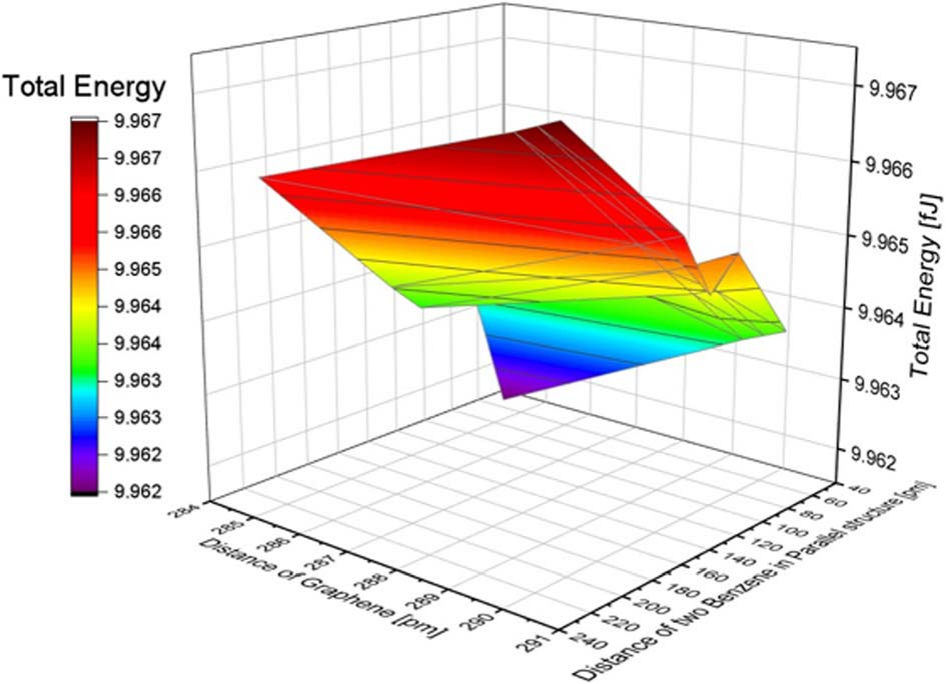
The effect of the distance difference between graphene and two benzene molecules in an inline structure.

In the simulation with two nonpolar molecules, this study primarily examined the effect of the distance between two molecules and the distance between two layers of graphene on the system's energy in both inline and stacked structures. In the inline structure, the definition of the distance between two molecules is measured from the right side of the left molecule to the right side of the right molecule. To investigate the effect of the distance between molecules, we varied the distance between two benzene molecules from 20 to 220 pm. The distance between graphene layers was set to 290.40, 290.00, 289.60, and 284.60 pm. According to Table 2, when the distance between the two molecules is less than or equal to 40 pm, the calculation results indicate that the molecules are too close for the system to be calculated accurately. The lowest energy occurs around 60 pm in our calculation model (see Fig. S5†). We conducted a full energy calculation by varying the distance between two inline benzene molecules and the spacing of graphene layers. This allows us to estimate which position on the CNT is the most stable for adsorption. The difference in distance between the two benzene molecules has a greater effect on the total energy than the difference in the distance between graphene layers, which has nearly no effect. The minimum energy in an inline structure is 9.962 fJ with a distance of 60 pm between benzene molecules and a distance of 284.6 pm between the two graphene layers, as shown in Fig. 4. Considering the effect of graphene, the distance differences between graphene layers have minimal impact on the total results, and the lowest energy, in this case, is 9.941 fJ when the two benzene molecules are located along the symmetry *x*-axis at 54.5 pm and the distance between graphene layers is 284.60 pm.

**Table 2:** Total energy changes with the distance of benzene molecules

Distance between two benzenes in an inline structure [pm]	Total energy [fJ]
0	Atoms are too close
20	Atoms are too close
40	Atoms are too close
60	9.964
80	9.964
100	9.965
120	9.965
140	9.965
160	9.966
180	9.966
200	9.967
220	9.967

### Benzene stack structure

We computed the total energy for a structure where two benzene molecules are stacked and positioned between graphene layers. The interplanar distance between benzene molecules and the distance between the graphene and benzene surfaces were treated as variables. We performed calculations with a distance of 60-220 pm between benzene molecules and 435.6, 435.0, and 434.4 pm between graphene layers. The results are listed in Table 3. The energy difference due to the distance between graphene layers was relatively small compared to the variation in distance between benzene molecules. As a result, the energy was minimized at 9.873 fJ when the distance between benzene molecules was 60 pm and the distance between graphene layers was 435.6 pm.

**Table 3:** Total energy changes with the distance of benzene molecules in a stack structure

Distance between two benzenes in a stack structure [pm]	Total energy [fJ]
60	9.873
100	9.964
120	9.973
140	9.975
180	9.973
220	9.955

**Fig. 5:**
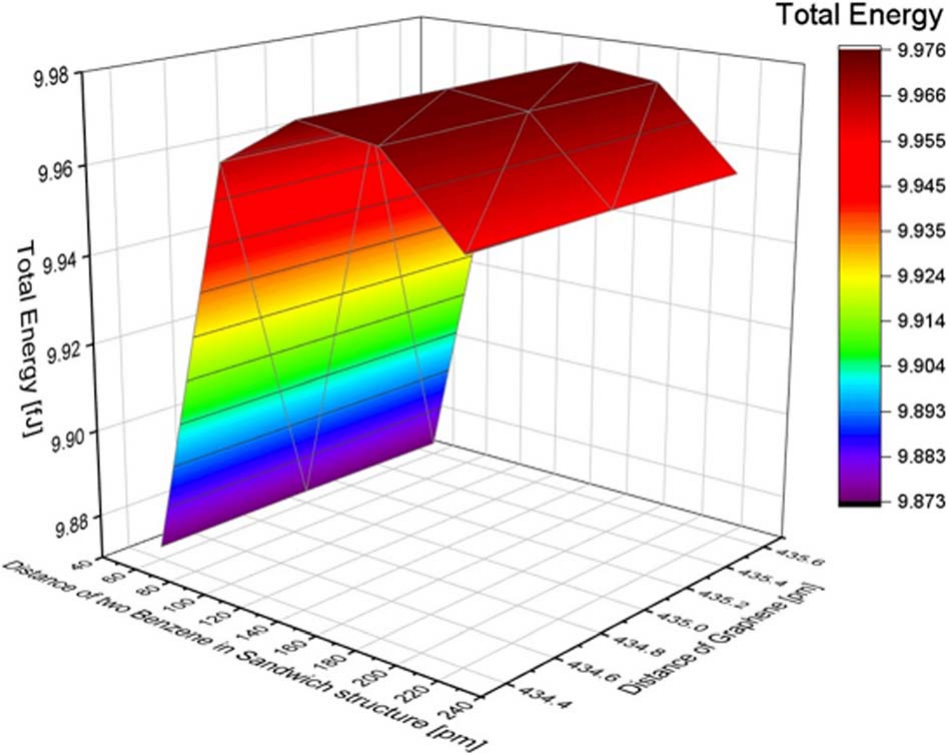
The effect of the distance difference between graphene and two benzene molecules in a stack structure.

Additionally, changes in the total energy with the position of two benzene molecules and the distance between graphene layers in the stacked structure were also examined, similar to the one benzene molecule model and benzene in the inline structure. We used the symmetry *x*-axis as a reference and adjusted the position of the two molecules from the centre to the edge of the graphene, as shown in Fig. 5. The position values of the symmetry *x*-axis are 0, 108.9, 217.8, 326.7, 471.9, and 617.1 pm (detailed structures are shown in Fig. S6 of the ESI†). In terms of the effect of graphene, the distance differences between graphene layers have less impact on the total results, and the lowest energy in this condition is 9.949 fJ. This is achieved when the two benzene molecules are located at a symmetry *x*-axis of 108.9 pm and the distance between graphene layers is 334.4 pm. The total energy of the benzene structure was calculated and is presented in Table 4. By comparing the total energy of the inline and stacked structures, we found that the lowest total energy obtained was 9.873 fJ with a distance of 60 pm between benzene molecules and a distance of 435.6 pm between the two layers of graphene. This suggests that benzene molecules adsorbed onto graphene in a stacked structure are more stable and require less energy than inline structures.

**Table 4:** Summary of total energy in the benzene structure^a^

Benzene model	One benzene without graphene	Two benzenes	
		Inline structure	Stack structure
Distance between two benzene in an inline structure [pm]	—	60	60
Distance between two layers of graphene in an inline structure [pm]		284.6	435.6
The lowest total energy [fJ]	1.005	9.962	9.873

a — represents there was no valid value.

### Total energy of one polar molecule

Methanol (MeOH) was used as the simulation model for polar molecules. The total energy of one methanol molecule, calculated using Gaussian 16, is 0.500 fJ.

### MeOH inline structure

**Fig. 6:**
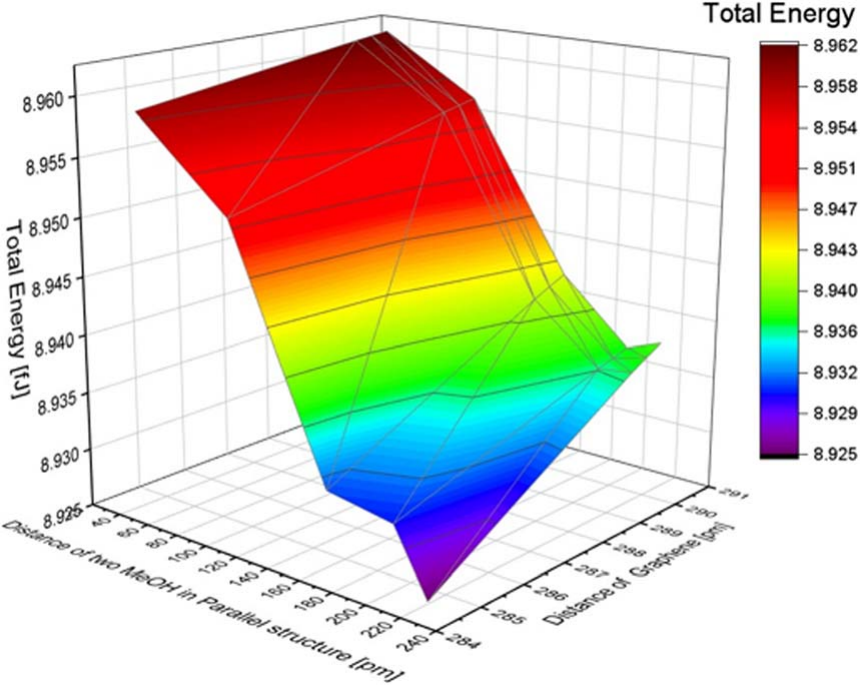
The effect of the distance difference between graphene and two MeOH molecules in an inline structure.

In this research, the distance between two MeOH molecules was defined as the distance from the carbon atom of the left molecule to the right hydrogen atom of the left molecule, as shown in Fig. S7 of the ESI.† To investigate the effect of the distance between the molecules, we varied the distance between the two MeOH molecules from 20 to 220 pm. In this simulation, structural relaxation of methanol molecules was not taken into account. Table 5 suggests that when the distance between two molecules is less than 20 pm, the calculation results indicate that the atoms are too close, and it is not possible to calculate the total energy. To investigate the trend of total energy concerning the distance between the two graphene sheets, we calculated the energy at different distances (see Fig. S8 of the ESI†). The lowest energy occurs at a distance of 200 pm, with a value of 8.937 fJ in our calculation model. The distance between graphene was varied to 290.40, 290.00, 289.60, and 284.60 pm. The change in total energy with the distance between the two layers of graphene in the inline structure of benzene is similar to the total energy change in the benzene molecule model. When the distance of graphene changes, the nearer distance showed lower energy. The distance difference between the two MeOH molecules has a greater effect on total energy than the distance difference from graphene, as reported in Fig. 6. The minimum energy in the inline structure is 8.925 fJ when the distance between the two MeOH molecules is 200 pm, and the distance between the two layers of graphene sheets is 284.6 pm.

**Table 5:** Total Energy changes with the distance of MeOH molecules in an inline structure

Distance between two MeOH in an inline structure [pm]	Total energy [fJ]
0	Atoms are too close
20	8.959
40	8.962
60	8.962
80	8.960
100	8.957
120	8.950
140	8.948
160	8.942
180	8.939
200	8.937
220	8.938

### MeOH stack structure

**Fig. 7:**
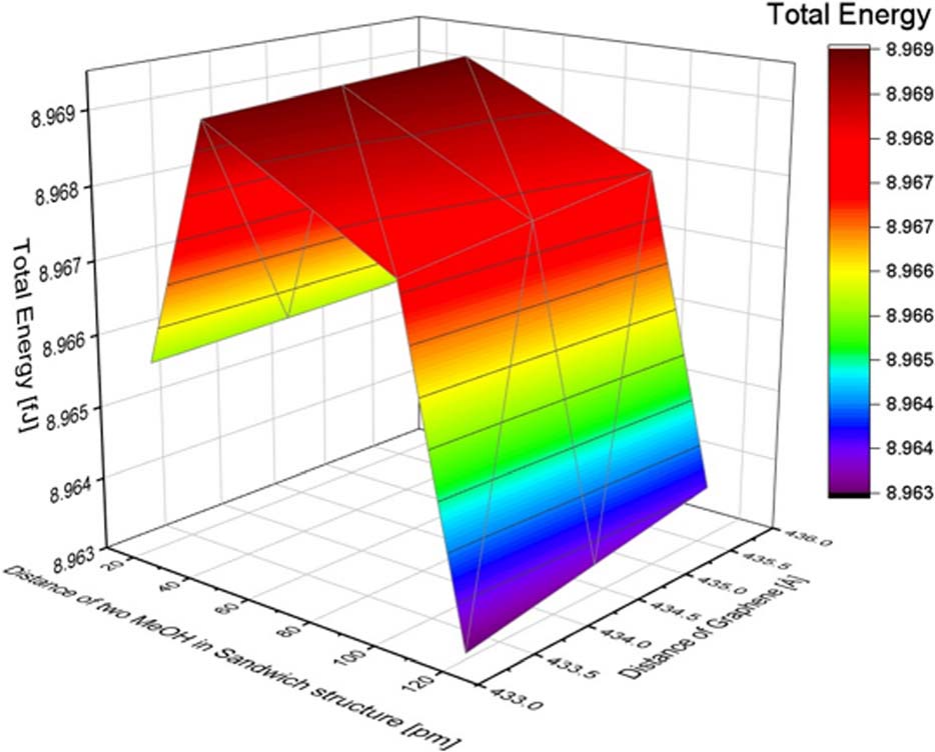
The effect of the distance difference between graphene and two MeOH molecules in a stack structure.

The distance between two molecules in a stack structure was defined as the distance between the planes of two carbon atoms (as shown in Fig. S9 of the ESI†). To investigate the effect of the distance, we varied the distance between two MeOH molecules. Based on the MeOH inline structure results, if the distance between two molecules is less than 20 pm, the calculation results indicate that the atoms are too close, making it impossible to calculate the total energy. Therefore, the distances in the simulation were set at 20, 40, 100, and 120 pm. The distance between graphene layers was adjusted to 435.6, 434.4, and 433.2 pm. The total energy changes with the distance of MeOH molecules in the stack structure are shown in Table 6. The total energy change with the distance between the two layers of graphene sheets in the MeOH stack structure is also presented. As a result, the total energy shows only a slight difference when the distance of graphene changes, although a shorter distance resulted in lower energy. To further illustrate the impact of the distance between the two methanol molecules and the distance between graphene layers in the stack structure, we refer to Fig. 7. The minimum energy in the MeOH stack structure is 8.963 fJ with a distance of 120 pm between the two methanol molecules and 433.2 pm between the two layers of graphene. The total energy of the methanol structure is presented in Table 7. By comparing the total energy of the inline and stack structures, we found that the lowest total energy obtained was 8.925 fJ with a distance of 220 pm between methanol molecules and 284.6 pm between the two layers of graphene. This suggests that MeOH molecules adsorbed onto graphene in an inline structure are more stable.

**Table 6:** Total Energy changes with the distance of MeOH Molecules in a stack structure

Distance between two MeOH in a stack structure [pm]	Total energy [fJ]
20	8.966
40	8.969
100	8.968
120	8.963

**Table 7:** Summary of total energy in the methanol structure^a^

Methanol model	One MeOH without graphene	Two MeOH	
		Inline structure	Stack structure
Distance between two MeOH in an inline structure [pm]	—	220	120
Distance between two layers of graphene in an inline structure [pm]		284.6	433.2
The lowest total energy [fJ]	0.500	8.925	8.963

a — represents there was no valid value.

In this study, the stabilization of polar and nonpolar molecules adsorbed on two graphene sheets, which simulate carbon nanotubes (CNTs), was computationally investigated using Gaussian 16. The results revealed that nonpolar molecules tend to adsorb in a stacked configuration with two molecules, while polar molecules preferentially adsorb in an inline structure with molecules aligned in a row, suggesting greater stability. Previous studies have shown that the mechanism by which the electrical resistance of CNT films changes upon molecular adsorption is due to localized variation in the dielectric constant at the CNT interface, altering the energy required for electron mobility. It is conceivable that adsorption in a stacked configuration can significantly influence the variation in the dielectric constant. Specifically, in the case of nonpolar molecules, as the gas concentration increases and multilayer adsorption becomes prevalent, it is expected that two molecules would adhere to the CNT interface, leading to adsorption isotherms exhibiting a Type VI multilayer adsorption curve according to the IUPAC classification. On the other hand, with polar molecules, it is preferable for them to adsorb in a linear arrangement, causing subsequent molecules beyond the first one to be positioned farther away from the CNT interface. Consequently, the influence on the localized dielectric constant variation becomes negligible, and as a result, the change in electrical resistance exhibits a monotonically increasing single-stage curve in response to gas concentration, akin to Type I behaviour as classified by IUPAC.

The remaining question is why adsorption isotherms showing three or more steps are not obtained in the adsorption of nonpolar molecules. In IUPAC Type VI, multiple steps are fundamental, and it does not limit adsorption to two steps. We considered that in a state where three or more molecules are stacked, there was not sufficient space in the interstices of the CNT interface when considering intermolecular distances. Therefore, for adsorption to occur in such a state, it would require very weak adsorption while being distant from the interface. At this point, the electrical effect decreases with the square of the distance, similar to the reason why the influence of the second and subsequent adsorbed molecules can be ignored in polar molecules. Thus, we suppose that adsorption isotherms showing three or more steps do not appear for the same reason.

## Conclusions

The research simulated the adsorption behaviour of polar and nonpolar molecules using a computational model. Based on the model results, two hypotheses regarding the adsorption mechanisms of these molecules were proposed. Gaussian 16 was employed to calculate the total energy of each possible structure. The study primarily focused on changes in total energy resulting from variations in the distance between graphene and molecules in both stack and inline structures. While nonpolar molecules tend to adsorb in a stacked configuration on two graphene interfaces, the results showed that inline adsorption is preferable for polar molecules. These findings suggest that similar adsorption behaviour may occur at CNT interfaces. Inline adsorption has a minimal effect on the local dielectric constant of the interface, which could explain why multiple-step adsorption isotherms are not observed.

## Data availability

The raw data required to reproduce these findings are available from the corresponding author on reasonable request.

## Author contributions

Mengli Zhang: data curation, investigation, writing – original draft. Shuhei Inoue: conceptualization, formal analysis, writing – original draft, supervision, investigation, funding acquisition. Yukihiko Matsumura: conceptualization, supervision, resources.

## Conflicts of interest

There are no conflicts to declare.

## Supplementary Material

RA-014-D4RA04474F-s001
