# Supplementary material for: Mechanism simulation of polar and nonpolar organic solvent vapor adsorption on a multiwall carbon nanotubes paper gas sensor
Source: RSC Adv. 2024 Aug 9;14(34):24985–91. doi: 10.1039/d4ra04474f (PMC11310837; doi:10.1039/d4ra04474f)
Supplement: RA-014-D4RA04474F-s001 [file RA-014-D4RA04474F-s001.pdf]

This journal is © The Royal Society of Chemistry 2024

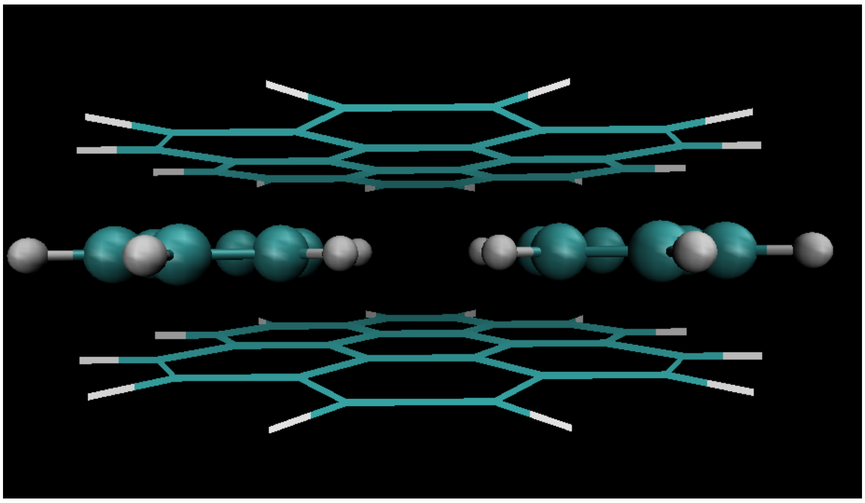

Figure S1. inline structure.

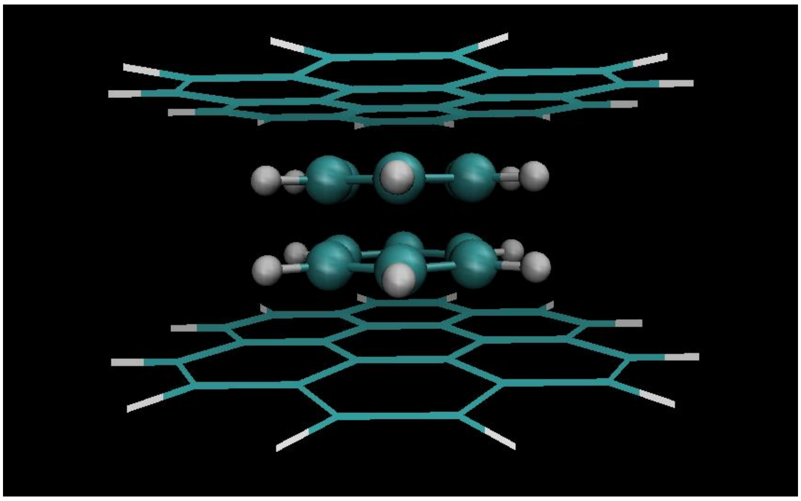

Figure S2. stack structure.

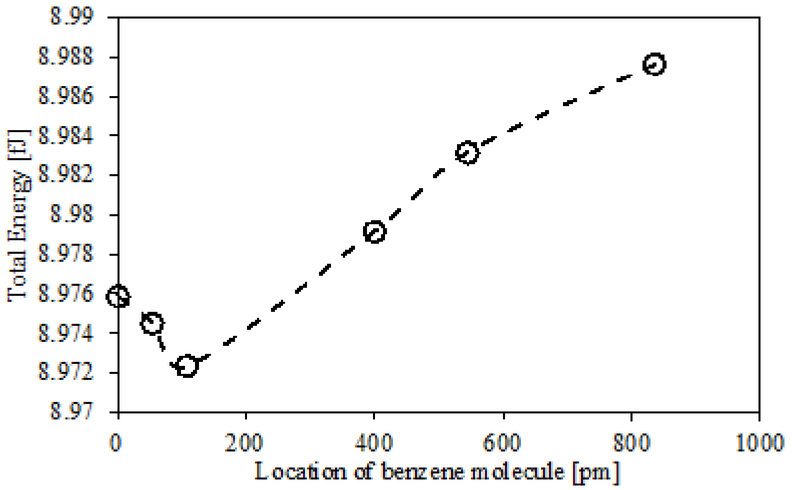

Figure S3. Energy changes with the location of one benzene from the center to the edge of the graphene.

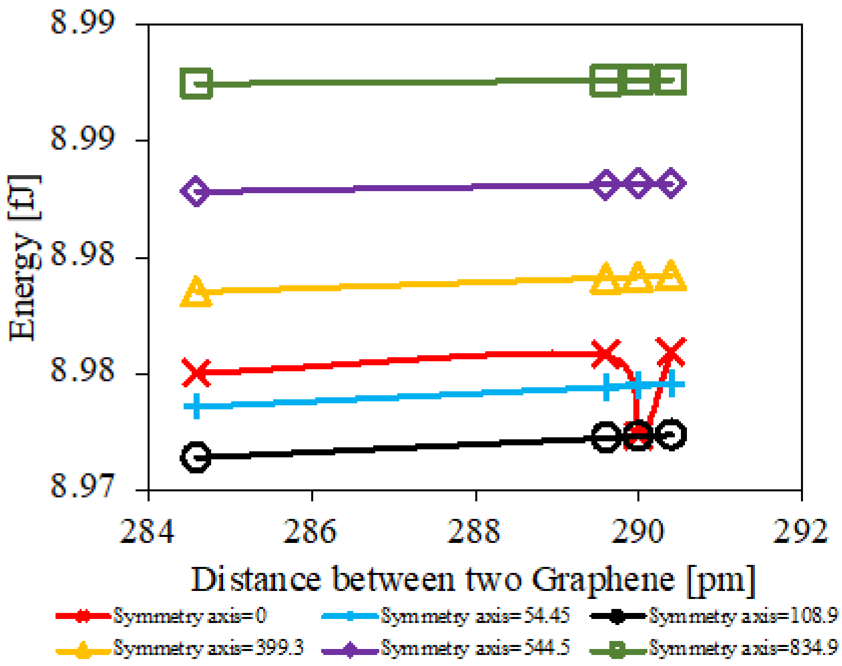

Figure S4. Energy changes with the distance of graphene for one benzene molecule model.

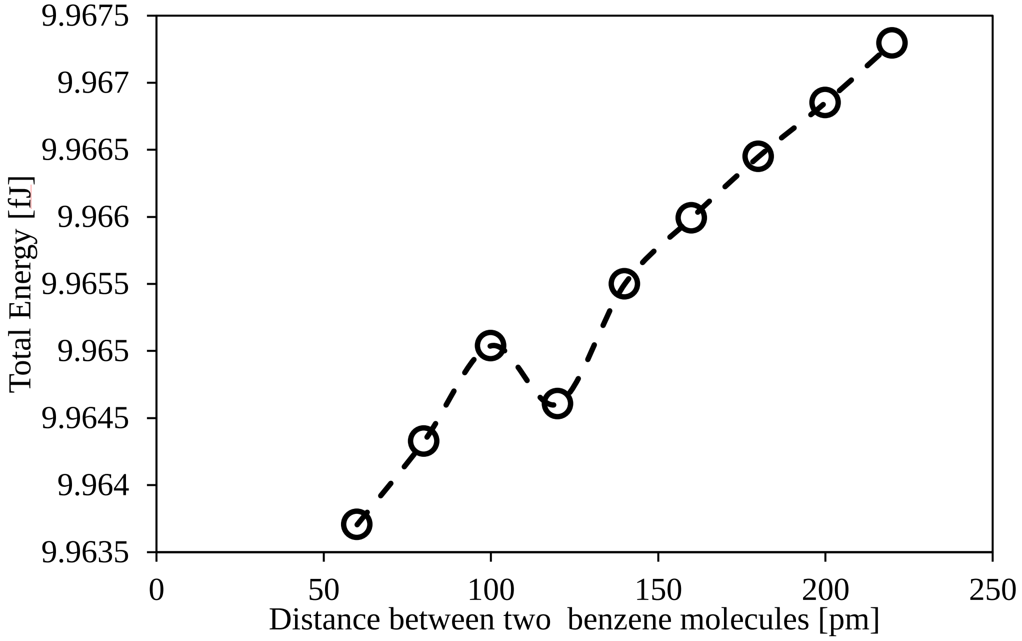

Figure S5. The trend of the total energy concerning the distance between the two benzene molecules in an inline structure.

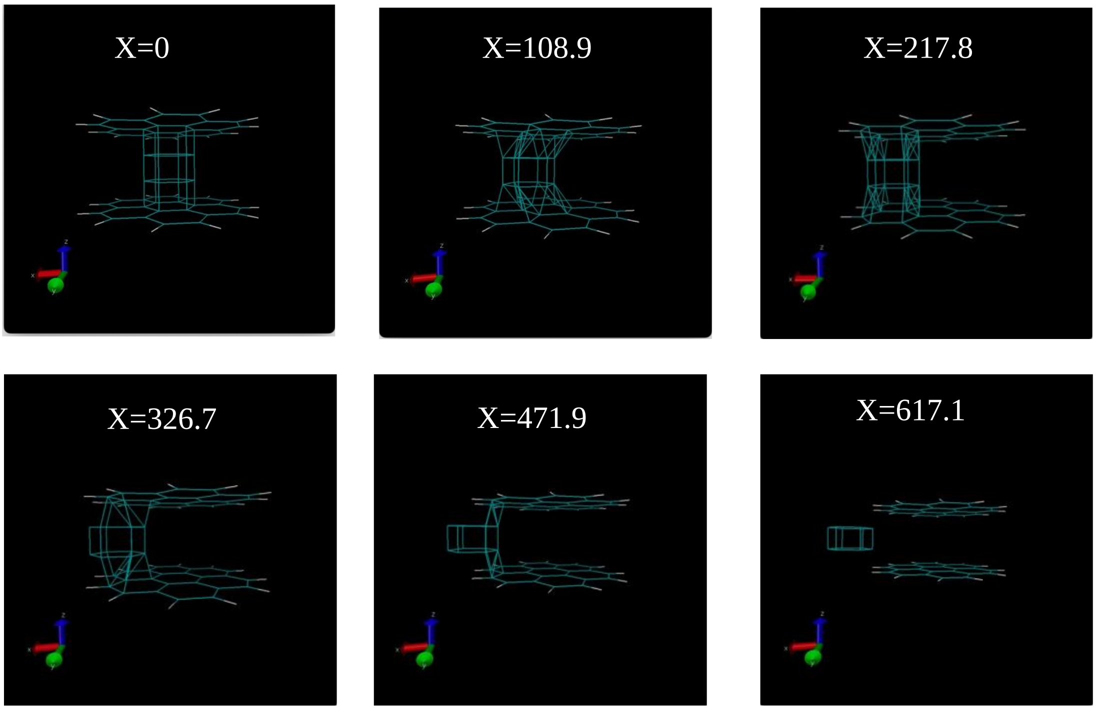

Figure S6. The position of two benzene molecules changes from the center to the edge of the graphene with the value of the symmetry x-axis 0, 108.9, 217.8, 326.7, 471.9, and 617.1 pm in a stack structure.
(X in this figure represents the symmetry axis of benzene)

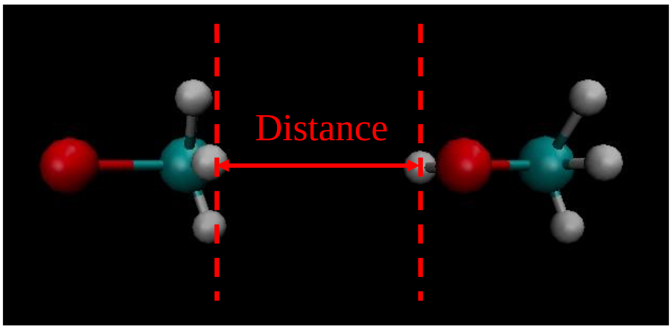

Figure S7. The definition of distance between two MeOH molecules in an inline structure.

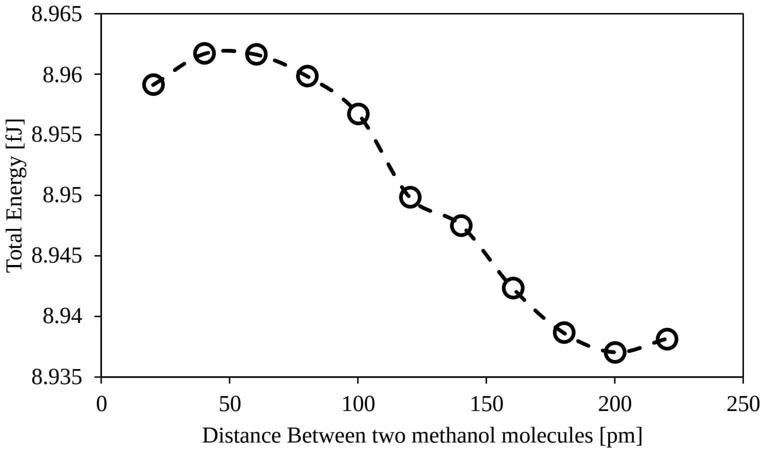

Figure S8. The trend of the total energy concerning the distance between the two MeOH molecules in an inline structure.

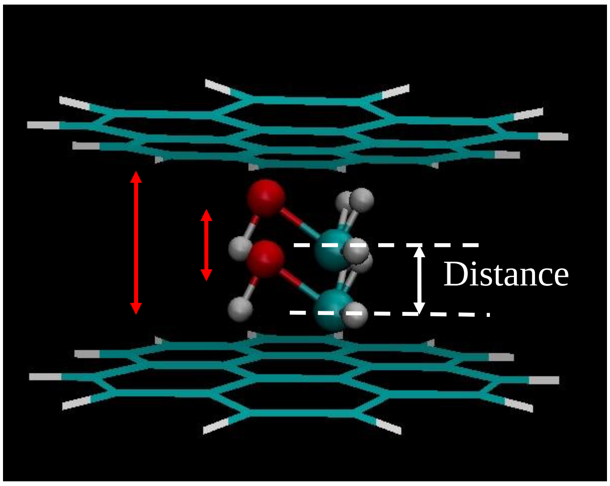

Figure S9. The definition of distance between two MeOH molecules in a stack structure and the red arrow represents the possible moving direction of molecules and graphene sheets.
